# Lepocreadiidae Odhner, 1905 and Aephnidiogenidae Yamaguti, 1934 (Digenea: Lepocreadioidea) of fishes from Moreton Bay, Queensland, Australia, with the erection of a new family and genus

**DOI:** 10.1007/s11230-018-9803-3

**Published:** 2018-05-31

**Authors:** Rodney A. Bray, Thomas H. Cribb, Scott C. Cutmore

**Affiliations:** 1 Department of Life Sciences, Natural History Museum, Cromwell Road, London, SW7 5BD UK; 2 School of Biological Sciences, The University of Queensland, St Lucia, QLD 4072 Australia

## Abstract

Digeneans of the lepocreadioid families Lepocreadiidae Odhner, 1905 and Aephnidiogenidae Yamaguti, 1934 from Moreton Bay, off southern Queensland, Australia, are recorded, along with the erection of a new family, Gibsonivermidae. Molecular data were generated for all representatives of these families collected during this study and a phylogram for members of the superfamily was generated based on the partial 28S rDNA dataset, placing these species in context with those previously sequenced. This phylogenetic analysis demonstrates that the monotypic *Gibsonivermis* Bray, Cribb & Barker, 1997 is isolated from all other lepocreadioids and supports the erection of Gibsonivermidae n. fam., which is defined morphologically, based particularly on the uniquely elongated male terminal genitalia, the distribution of the uterus in the forebody and the presence of a uroproct. *Mobahincia teirae* n. g., n. sp. is reported from *Platax teira* (Forsskål) in Moreton Bay and off Heron Island and New Caledonia. Recognition of this new genus is based on molecular results and the combination of caeca abutting the posterior body wall and the lack of an anterior body scoop or flanges. The following lepocreadioid species are reported from Moreton Bay for the first time: *Bianium arabicum* Sey, 1996 in *Lagocephalus lunaris* (Bloch & Schneider), *Diploproctodaeum* cf. *monstrosum* Bray, Cribb & Justine, 2010 in *Arothron hispidus* (Linnaeus), *Multitestis magnacetabulum* Mamaev, 1970 and *Neomultitestis aspidogastriformis* Bray & Cribb, 2003 in *Platax teira* and *Opechona austrobacillaris* Bray & Cribb, 1998 in *Pomatomus saltatrix* (Linnaeus). *Bianium plicitum* (Linton, 1928) is reported from *Torquigener squamicauda* (Ogilby) for the first time. Sequences of newly collected specimens of *Austroholorchis sprenti* (Gibson, 1987) indicate that the species forms a clade with other members of the Aephnidiogenidae, agreeing with its morphology. The phylogenetic status of all newly sequenced species is discussed.

**Electronic supplementary material:**

The online version of this article (10.1007/s11230-018-9803-3) contains supplementary material, which is available to authorized users.

## Introduction

During January and July 2016, workshops were held at the Moreton Bay Research Station at Dunwich on North Stradbroke Island, off southern Queensland, Australia, as part of a collaborative study of the metazoan parasite fauna of the fishes, particularly the commercially important fishes, of Moreton Bay. The present work is a report on some of the digeneans found, framed as an overview of our knowledge of the closely related families Lepocreadiidae Odhner, 1905 and Aephnidiogenidae Yamaguti, 1934 in Moreton Bay. The lepocreadioid fauna of Australian and other Indo-Pacific fishes has been ‘subjected to recent sustained study’ (Cribb & Bray, 2011). This has been documented in some 31 articles (see Bray et al., 2009, and references therein; Bray et al., 2010b; Bray et al., 2010a); however, much work remains to be done. Some genera are large and/or complex and require molecular data to elucidate their status.

Bray & Cribb (2012) divided members of the Lepocreadiidae Odhner, 1905 as recognised by Bray (2005) into three families based on a molecular phylogeny. These three families, the Lepocreadiidae, Lepidapedidae Yamaguti, 1958 and Aephnidiogenidae Yamaguti, 1934, had previously been considered subfamilies of the Lepocreadiidae (see Bray, 2005). In this paper, we analyse species of two of these three families found in Moreton Bay. A new family and a new genus and species are erected. In addition, this report summarises information from earlier studies in the region. Collections representing specimens of two lepocreadiid genera (*Lepotrema* Ozaki, 1932 and *Preptetos* Pritchard, 1960) and one lepidapedid genus (*Postlepidapedon* Zdzitowiecki, 1993) will be incorporated in genus-specific studies later and are thus not reported here. Novel 28S and ITS2 rDNA sequences are reported for all new collections, which enable the placement of many of the Moreton Bay species in a wider phylogenetic context.

## Materials and methods

*Specimen collection and morphological analysis* Fish were collected by line-fishing, spear-fishing, seine netting and from the commercial tunnel-net fishery in Moreton Bay, Queensland, Australia. Fish were euthanised and examined for trematodes, as described by Cribb & Bray (2010). Those collected were fixed by pipetting into near-boiling saline and immediately preserved in formalin or 70% ethanol. Whole-mounts were stained with Mayer’s paracarmine or Mayer’s haematoxylin, dehydrated in a graded ethanol series, cleared in beechwood creosote or methyl salicylate and mounted in Canada balsam. Measurements were made through a drawing tube on an Olympus BH-2 microscope, using a Digicad Plus digitising tablet and Carl Zeiss KS100 software adapted by Imaging Associates, and are quoted in micrometres, with the range and the mean in parentheses. The following abbreviations are used: NHMUK, Natural History Museum, London, UK; MNHN, Museum National d’Histoire Naturelle, Paris, France; QM, Queensland Museum Collection, Brisbane, Australia.

*Molecular sequencing and phylogenetic analysis*

Specimens for molecular analysis were processed according to the protocols used by Sun et al. (2014). The complete ITS2 rDNA region was amplified and sequenced using the primers 3S (Morgan & Blair, 1995) and ITS2.2 (Cribb et al., 1998) and the partial D1-D3 28S rDNA region using LSU5 (Littlewood, 1994), 300F (Littlewood et al., 2000), ECD2 (Littlewood et al., 1997) and 1500R (Snyder & Tkach, 2001). Geneious® version 10.2.3 (Kearse et al., 2012) was used to assemble and edit contiguous sequences and the start and end of the ITS2 rDNA region were determined by annotation through the ITS2 Database (Keller et al., 2009; Ankenbrand et al., 2015) using the ‘Metazoa’ model.

The partial 28S rDNA sequences generated during this study were aligned with sequences of related species of the Lepocreadioidea Odhner, 1905 from GenBank using MUSCLE version 3.7 (Edgar 2004) run on the CIPRES portal (Miller et al., 2010), with ClustalW sequence weighting and UPGMA clustering for iterations 1 and 2. The resultant alignment was refined by eye using MESQUITE (Maddison & Maddison, 2017). The ends of each sequence were trimmed, and ambiguously aligned regions were identified and masked manually (those constituting more than three bases and present in greater than 5% of the sequences in the dataset).

Bayesian inference and maximum likelihood analyses of the 28S dataset were conducted to explore relationships among these taxa. Bayesian inference analysis was performed using MrBayes version 3.2.6 (Ronquist et al., 2012) and maximum likelihood analysis using RAxML version 8.2.10 (Stamatakis, 2014), both run on the CIPRES portal. The best nucleotide substitution model was estimated using jModelTest version 2.1.10 (Darriba et al., 2012). Both the Akaike Information Criterion (AIC) and Bayesian Information Criterion (BIC) predicted the GTR+I+Γ model as the best estimator; Bayesian inference and maximum likelihood analyses were conducted using the closest approximation to this model. Nodal support in the maximum likelihood analysis was estimated by performing 100 bootstrap pseudoreplicates. Bayesian inference analysis was run over 10,000,000 generations (ngen = 10,000,000) with two runs each containing four simultaneous Markov Chain Monte Carlo (MCMC) chains (nchains = 4) and every 1,000th tree saved. Bayesian inference analysis used the following parameters: nst = 6, rates = invgamma, ngammacat = 4, and the priors parameters of the combined dataset were set to ratepr = variable. Samples of substitution model parameters and tree and branch lengths were summarised using the parameters: sump burnin = 3,000 and sumt burnin = 3,000. Species of the families Cryptogonimidae Ward, 1917 and Apocreadiidae Skrjabin, 1942 were designated as functional outgroup taxa, *sensu* Bray et al. (2009).

**Family Lepocreadiidae Odhner, 1905**

**Subfamily Lepocreadiinae Odhner, 1905**

**Genus** ***Bianium*** **Stunkard, 1930**

***Bianium plicitum*** **(Linton, 1928) Stunkard, 1931**

Syn. *Psilostomum plicitum* Linton, 1928

*Type-host*: *Larus argentatus* Pontoppidan (Charadriiformes: Laridae), herring gull.

*Type-locality*: Woods Hole, Massachusetts, USA.

New records:

*Hosts*: *Torquigener squamicauda* (Ogilby), brush-tail toadfish; *T*. *pleurogramma* (Regan), weeping toado (Tetraodontiformes: Tetraodontidae).

*Localities*: Ex *T. squamicauda*, Moreton Banks, Moreton Bay (27°24′S, 153°20′E); ex *T. pleurogramma*, off Amity, Moreton Bay (27°24′S, 153°26′E).

*Site in host*: Intestine.

*Voucher material*: Three specimens in the QM G237251–3, one in the NHMUK 2018.3.26.1.

*Representative DNA sequences*: ITS2 rDNA, four identical replicates (two in GenBank MH157055-MH157056); 28S rDNA, one sequence (GenBank MH157066).

*New measurements*: Supplementary Table S1.

### Remarks

**Fig. 1:**
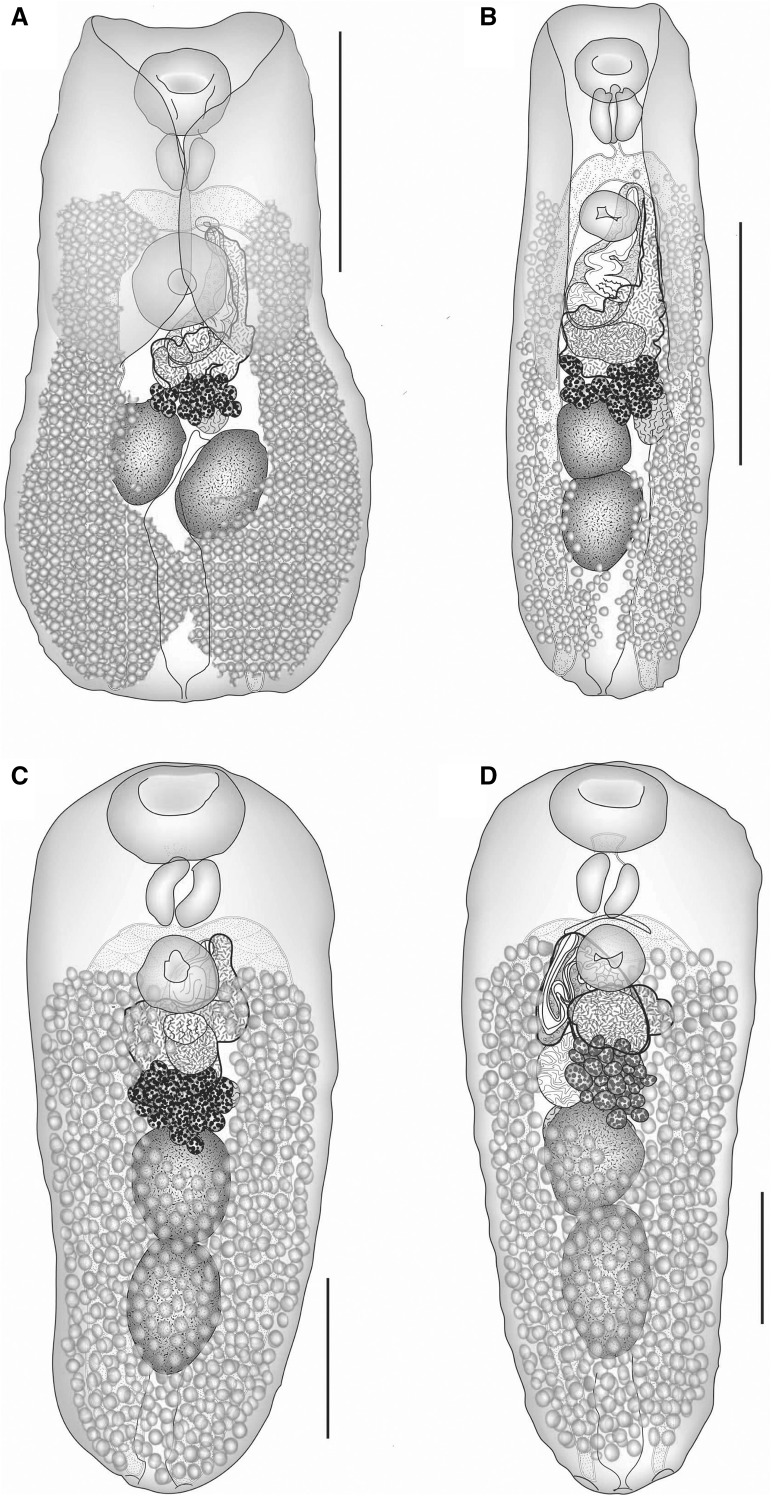
A, *Bianium plicitum* (Linton, 1928) ex *Torquigener squamicauda*, ventral view, uterus in outline; B, *Bianium arabicum* Sey, 1996 ex *Lagocephalus lunaris*, ventral view, uterus in outline; C, *Mobahincia teirae* n. g., n. sp. ex *Platax teira*, Moreton Bay, ventral view, uterus in outline; D, *Mobahincia teirae* n. g., n. sp. ex *Platax teira*, off Heron Island, ventral view, uterus in outline. *Scale-bars*: A, B, 500 μm; C, D, 200 μm

**Fig. 2:**
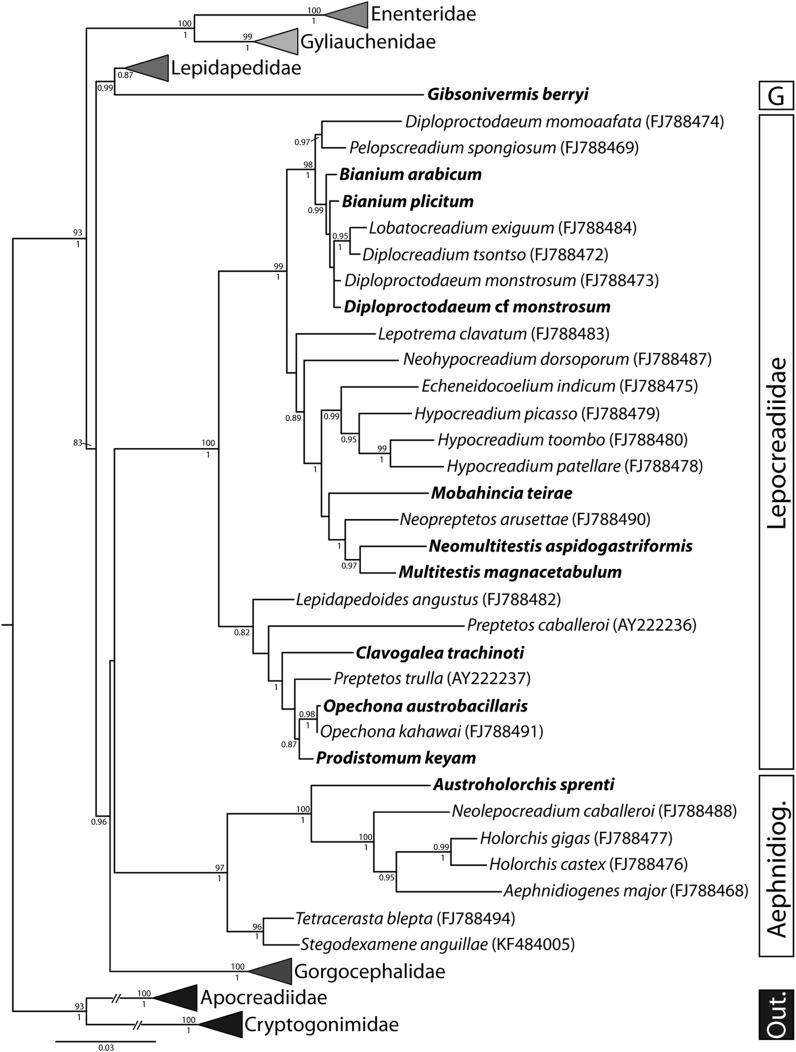
Relationships between members of the seven families of the superfamily Lepocreadioidea based on maximum likelihood analysis of the partial 28S rDNA dataset. Species from Moreton Bay are shown in bold and clades representing the Enenteridae, Gorgocephalidae, Gyliauchenidae and Lepidapedidae are collapsed for brevity. Maximum likelihood bootstrap support values are shown above the nodes and Bayesian inference posterior probabilities below. Support values < 80 and < 0.80 are not shown. Outgroup taxa are species of the Apocreadiidae and Cryptogonimidae. *Abbreviations*: Aephnidiog., Aephnidiogenidae; G, Gibsonivermidae; Out., outgroup taxa

The new specimens (Fig. 1A) are morphologically identical to those reported from Moreton Bay by Bray & Cribb (1998) from Whitley’s toadfish *Torquigener whitleyi* (Paradice) and *T. pleurogramma*. New ITS2 rDNA sequences of specimens from *T. squamicauda* and *T. pleurogramma* were identical. Analysis of the 28S data showed that this species forms a strongly supported clade with similar lepocreadiid species from tetraodontiforms (other species of *Bianium*, *Pelopscreadium* Dronen, Blend, Khalifa, Mohamadain & Karer, 2016, *Diplocreadium* Park, 1939, *Diploproctodaeum* La Rue, 1926 and *Lobatocreadium* Madhavi, 1972); nodal support for relationships within this clade was weak (Fig. 2). The two species of *Bianium* included in the phylogenetic analyses are paraphyletic with respect to species of *Diplocreadium*, *Diploproctodaeum* and *Lobatocreadium*. The status of these specimens from Moreton Bay as identical to *Bianum plicitum* as described by Linton (1928) is yet to be tested by DNA sequence comparison, and we think it highly likely that forms from eastern Australian waters are not conspecific with the original specimens from off north-eastern USA.

***Bianium arabicum*** **Sey, 1996**

*Type-host*: *Lagocephalus lunaris* (Bloch & Schneider) (Tetraodontiformes: Tetraodontidae), lunartail puffer.

*Type-locality*: Off Kuwait, Arabian Gulf.

New records:

*Host*: *Lagocephalus lunaris*.

*Locality*: Off Wynnum North, Moreton Bay (27°23′S, 153°11′E).

*Site in host*: Intestine.

*Voucher material*: Two specimens in the QM G237254–5, one in the NHMUK 2018.3.26.2.

*Representative DNA sequences*: ITS2 rDNA, one sequence (GenBank MH157054); 28S rDNA, one sequence (GenBank MH157076).

*New measurements*: Supplementary Table S1.

### Remarks

In the original description of *B. arabicum*, Sey (1996) stated ‘Along the lateral sides of body longitudinal folds present, bending ventrally and ending at posterior extremity’. The longitudinal folds (flanges) do not appear to reach the full length of the body in our specimens (Fig. 1B). When describing specimens considered to be this species from the silverstripe blaasop *Lagocephalus sceleratus* (Gmelin) off New Caledonia, Bray et al. (2010a) said ‘it has full-length lateral folds of the body (or scoop-sides), although the full extent is not always visible on both sides of the worm’. These authors pointed out the similarity of these worms to those described from *L. lunaris* by Hafeezullah (1970) under the name *B. plicitum* (Linton, 1928) from off Chennai (as Madras) in the Bay of Bengal and by Shen & Tong (1990) under the name *B. dayawanense* Shen & Tong, 1990 from Daya Bay, China [in this case the host is quoted as *L. lunaris spadiceus* (Richardson)]. The lateral flanges of the Bay of Bengal worms are similar to those seen in our specimens and the dimensions are close to those found by Sey (1996) and Bray et al. (2010a) (Supplementary Table S1). The Chinese worms tend to be larger and the flanges are illustrated as distinct flaps reaching only to the ventral sucker level. It is not possible to be certain of the status of all these forms, but it appears that the Bay of Bengal specimens are more similar to the worms here considered *B. arabicum*. This is the first report of *B. arabicum* from Moreton Bay.

Analysis of the 28S data showed that this species forms a strongly-supported clade with similar lepocreadiid species from tetraodontiforms; nodal support for relationships within this clade were weak (Fig. 2) and, as discussed above, the two species of *Bianium* did not form a clade.

**Genus** ***Clavogalea*** **Bray, 1985**

***Clavogalea trachinoti*** **(Fischthal & Thomas, 1968) Bray & Gibson, 1990**

Syns *Stephanostomum trachinoti* Fischthal & Thomas, 1968; *Opechona pseudobacillaris* Fischthal & Thomas, 1970

*Type-host*: *Trachinotus goreensis* Cuvier (Perciformes: Carangidae), longfin pompano.

*Type-locality*: Off Iture, Elmina, Ghana.

New records:

*Host*: *Trachinotus coppingeri* Günther (Perciformes: Carangidae), swallowtail dart.

*Locality*: Off Green Island, Moreton Bay (27°25′S, 153°14′E).

*Site in host*: Intestine.

*Voucher material*: Six specimens in the QM G237275–80.

*Representative DNA sequences*: ITS2 rDNA, three replicates (one in GenBank MH157057); 28S rDNA, one sequence (GenBank MH157067).

### Remarks

Bray & Gibson (1990) redescribed the original specimens of *Stephanostomum trachinoti* Fischthal & Thomas, 1968 and its synonym *Opechona pseudobacillaris* Fischthal & Thomas, 1970, and placed the species in *Clavogalea*. Bray & Cribb (1998) redescribed the worm based on new material from the large-spotted dart *Trachinotus botla* (Shaw) off Heron Island (southern Great Barrier Reef) and *T. coppingeri* Günther off northern New South Wales and in Moreton Bay. Our newly collected material appears identical to these descriptions. New 28S rDNA data were identical to sequences reported by Bray et al. (2009) based on specimens from *T. coppingeri* collected off Heron Island. Phylogenetic analysis of the 28S dataset indicates that, of taxa available for analysis, this species is most closely related to *Preptetos trulla* (Linton, 1907), *Prodistomum keyam* Bray & Cribb, 1996, *Opechona austrobacillaris* Bray & Cribb, 1998 and *Opechona kahawai* Bray & Cribb, 2003. These five species formed a clade in the phylogenetic analysis with *C. trachinoti* as sister to a clade of the other four species; however, nodal support for this topology was poor (Fig. 2).

**Genus** ***Diplocreadium*** **Park, 1939**

***Diplocreadium tangaloomaense*** **Bray, Cribb & Barker, 1996 (emend.)**

*Type-host*: *Paramonacanthus japonicus* (Tilesius) (Tetraodontiformes: Monacanthidae), hairfinned leatherjacket.

*Type-locality*: Off Tangalooma, Moreton Bay, Queensland.

### Remark

This species has not been detected since its original description and no material is available for molecular characterisation.

***Diploproctodaeum monstrosum*** **Bray, Cribb & Justine, 2010**

*Type-host*: *Arothron stellatus* (Anonymous) (Tetraodontiformes: Tetraodontidae), stellate puffer.

*Type-locality*: Off Mermaid Beach, Lizard Island, Queensland, Australia.

***Diploproctodaeum*** **cf.** ***monstrosum***

New records

*Host*: *Arothron hispidus* (Linnaeus), white-spotted puffer.

*Locality*: Off Peel Island, Moreton Bay (27°30′S, 153°20′E).

*Site*: Intestine.

*Voucher material*: Three specimens in the QM G237281–3.

*Representative DNA sequences*: ITS2 rDNA, two replicates (one in GenBank MH157059); 28S rDNA, one sequence (GenBank MH157069).

### Remarks

Bray et al. (2010a) reported this species in *Arothron stellatus* and *A. mappa* from off Lizard Island. They pointed out that the sequence of ‘*Diploproctodaeum* sp.’ from *A. stellatus* off Lizard Island (GenBank FJ788474), used in the study of Bray et al. (2009), referred to this species. 28S sequence data generated from the new Moreton Bay material differs from that sequence by 5 bases. No morphological differences could be detected between the two collections, but only a relatively small number of specimens has been collected and the rather amorphous structure of these worms makes morphological comparisons difficult. Given that the two sites are only approximately 1,650 km apart, a 5 bp difference in the 28S rDNA raises the possibility of the presence or more than one species. However, we consider the current evidence insufficient to suggest that specimens from Moreton Bay represent a species distinct from that occurring on the northern Great Barrier Reef but consider the issue worthy of further consideration in the light of additional genetic data from more variable gene regions (Blasco-Costa et al., 2016). For the present, the designation *D.* cf. *monstrosum* seems the best way to draw attention to these issues.

***Diploproctodaeum yosogi*** **Bray, Cribb & Barker, 1996**

*Type-host*: *Paramonacanthus japonicus* (Tilesius) (Tetraodontiformes: Monacanthidae), hairfinned leatherjacket.

*Type-locality*: Off Mud Island, Moreton Bay, Queensland.

### Remark

This species has not been detected since its original description and no material is available for molecular characterisation.

**Genus** ***Lepocreadioides*** **Yamaguti, 1936**

Syn. *Bicaudum* Bilqees, 1971

***Lepocreadioides orientalis*** **Park, 1939**

*Type-host*: *Cynoglossus joyneri* Günther, red tonguesole (Pleuronectiformes: Cynoglossidae).

*Type-locality*: Off Simmi Island, North Tyôsen, Korea.

### Remark

This species has not been re-collected from Moreton Bay since the report from the fourlined tonguesole *Cynoglossus bilineatus* (Lacépède) by Bray & Cribb (1998) and no material is available for molecular characterisation.

**Genus** ***Mobahincia*** **n. g.**

*Diagnosis*

Body broader anteriorly, tapering posteriorly. Tegument spined. Eye-spot pigment scattered at pharyngeal level. Oral sucker transversely oval, subterminal. Ventral sucker rounded, smaller than oral sucker, in anterior quarter of body-length. Prepharynx short. Pharynx oval. Oesophagus not detected. Intestinal bifurcation dorsal to anterior part of ventral sucker or just in forebody. Caeca straight, reaching to posterior extremity where they abut body wall at base on small indentations; ani possibly present. Testes two, oval, entire, tandem contiguous, in mid-hindbody. External seminal vesicle large, saccular, dorsal to uterus. Cirrus-sac claviform. Internal seminal vesicle large, oval, curved. Pars prostatica oval vesicular, lined with anuclear cell-like bodies. Ejaculatory duct thick-walled muscular, long, complexly folded. Genital atrium small. Genital pore sinistral to antero-sinistral to ventral sucker. Ovary multilobate, immediately pre-testicular. Seminal receptacle canalicular. Mehlis’ gland dorsal to ovary. Uterus between ovary and ventral sucker, intracaecal. Eggs tanned, operculate. Vitellarium in follicular fields at ventral sucker level and in hindbody. Parasites in intestine of ephippid fishes.

*Type-species*: *Mobahincia teirae* n. sp.

*Etymology*: The generic name is a feminine noun derived from the localities at which this genus has been found: Moreton Bay (Moba), Heron Island (hi), New Caledonia (nc).

*ZooBank registration*: To comply with the regulations set out in article 8.5 of the amended 2012 version of the *International Code of Zoological Nomenclature* (ICZN, 2012), details of the new genus have been submitted to ZooBank. The Life Science Identifier (LSID) for *Mobahincia* n. g. is urn:lsid:zoobank.org:act:8543D3CA-81FC-43A7-9ACB-6BA5DE6D6BA4.

### Remarks

The species on which this new genus is based appears morphologically closely related to members of *Diploproctodaeum* and *Bianium* in having its caeca abutting the posterior body wall, giving the appearance of ani, the usual condition in species of the latter genera; however, there is no indication of an anterior scoop as is usually present in these taxa. Molecular evidence suggests unambiguously that the new genus is not closely related to members of these two genera. The exact relationship of this species is not well resolved in the phylogram derived from the 28S analyses of the currently available lepocreadiid sequences (many relationships within the family have poor support), but it is clear that it does not resolve within the well-supported clade which includes *Diploproctodaeum* and *Bianium* species (Fig. 2). Following the key to the Lepocreadiidae produced by Bray (2005), the species appears closest to members of *Lobatocreadium* or *Pseudocreadium* Layman, 1930; the new genus differs from both in the presence of long caeca abutting the body-wall and the terminal excretory pore. The vitellarium is more extensive in both species of *Lobatocreadium* and *Pseudocreadium,* and in members of the latter genus the testes are symmetrical. We conclude that the relationships of this form are best expressed by the erection of a new genus.

***Mobahincia teirae*** **n. sp.**

*Type-host*: *Platax teira* (Forsskål) (Perciformes: Ephippidae), longfin batfish.

*Type-locality*: Four Beacons, Moreton Bay (27°10′S, 153°21′E).

*Other localities*: Off Heron Island (23°27′S, 151°55′E); Nouméa Fish Market, New Caledonia.

*Site in host*: Intestine.

*Type-material*: Holotype QM G237256 and 12 paratypes QM G237257–60, NHMUK 2018.3.26.5–8.

*Voucher material*: Off Heron Island: QM G237261; off New Caledonia: MNHN JNC2872F.

*Representative DNA sequences*: ITS2 rDNA, five replicates (one in GenBank MH157058); 28S rDNA, one sequence (GenBank MH157068).

*ZooBank registration*: To comply with the regulations set out in article 8.5 of the amended 2012 version of the *International Code of Zoological Nomenclature* (ICZN, 2012), details of the new species have been submitted to ZooBank. The Life Science Identifier (LSID) for *Mobahincia teirae* n. sp. is urn:lsid:zoobank.org:act:7FD7D6C8-D114-4C5E-BEDA-E25CB81979E9.

*Etymology*: The specific epithet is derived from that of the host species.

### Description (Fig. 1C, D)

[Based on 7 ovigerous and seven non-ovigerous specimens from Moreton Bay, 1 specimen from off Heron Island and 1 specimen from off New Caledonia; measurements given in Table 1.] Body broader anteriorly, tapering posteriorly. Body spines small on anterior ‘shoulders’, much more robust along remainder of body, reach close to posterior extremity. Eye-spot pigment scattered at pharyngeal level. Oral sucker transversely oval, subterminal. Ventral sucker rounded, smaller than oral sucker, in anterior quarter of body-length. Prepharynx short, mainly in posterior concavity of oral sucker. Pharynx oval. Oesophagus not detected. Intestinal bifurcation dorsal to anterior part of ventral sucker or just in forebody. Caeca straight, reach to posterior extremity where they abut body wall at base on small indentations; ani possibly present.

**Table 1:** Measurements and ratios of *Mobahincia teirae* ex *Platax teira*

Locality	Moreton Bay (n = 7)		New Caledonia (n = 1)	Heron Island (n = 1)
	Range	Mean		
Body	685–1,018 × 344–427	834 × 383	750 × 395	1,013 × 415
Forebody length	186–234	204	214	221
Pre-oral lobe length	0–5	3	6	5
Oral sucker	104–141 × 148–190	125 × 170	127 × 179	115 × 170
Prepharynx length	0–35	5	0	8
Pharynx	82–100 × 80–105	90 × 89	107 × 97	84 × 86
Oesophagus length	0		18	24
Distance from intestinal bifurcation to ventral sucker (IB-VS)	0–16	4	0	0
Distance from vitellarium to ventral sucker	0	0	19	0
Ventral sucker	79–112 × 90–122	96 × 103	89 × 98	93 × 99
Cirrus-sac	129–189 × 71–90	158 × 79	121 × 44	175 × 56
Distance from external seminal vesicle to ventral sucker	85–118	102	70	169
Distance from ventral sucker to ovary (VS-Ov)	20–51	34	17	61
Ovary	84–115 × 112–169	102 × 134)	64 × 110	132 × 133
Distance from ovary to anterior testis	0	0	0	0
Anterior testis	118–144 × 123–147	128 × 131	112 × 141	163 × 137
Distance between testes	0	0	0	0
Posterior testis	106–214 × 107–137	157 × 121	125 × 144	209 × 129
Post-testicular distance	112–190	150	128	190
Post-caecal distance	0–25	5	0	0
Eggs	58–70 × 26–41	62 × 34	63 × 23	69 × 33
Width (%)	41.9–59.0	46.5	52.7	41.0
Forebody (%)	21.8–27.2	24.7	28.5	21.8
Sucker length ratio	1:0.67–0.90	1:0.77	1:0.70	1:0.81
Sucker width ratio	1:0.58–0.64	1:0.60	1:0.55	1:0.58
Oral sucker: pharynx width	1:1.72–2.09	1:1.92	1:1.84	1:1.98
Ventral sucker to ovary (%)	2.63–5.02	4.04	2.32	6.06
External seminal vesicle to ventral sucker as % of VS-Ov	283–418	351	403	274
Post-testicular distance (%)	16–20	18	17.1	18.8
Prepharynx (%)	0–16.4	2.34	0	3.51
Oesophagus (%)	0	0	2.37	2.37
Distance IB-VS (%)	0–1.76	0.50	0	0
Vitellarium to ventral sucker distance (%)	0	0	2.47	0
Ovary to anterior testis (%)	0	0	0	0
Distance between testes (%)	0	0	0	0
Cirrus-sac length (%)	16.4–22.0	19.0	16.2	17.2
Pre-vitelline distance	186–234	204	195	221
Pre-vitelline distance (%)	21.8–27.2	24.7	26.0	21.8
Oesophagus length as % of forebody length	0	0	8.33	10.9
Distance IB-VS as % of forebody length	0–7.31	2.02	0	0
Vitellarium to ventral sucker distance as % of forebody length	0	0	8.66	0

*Note*: (%), percent of body-length where not otherwise noted; IB-VS, intestinal bifurcation to ventral sucker distance. Where length is followed by width, the two are separated by an ‘×’

Testes 2, oval, entire, tandem contiguous, in mid-hindbody. External seminal vesicle large, saccular, dorsal to uterus. Cirrus-sac claviform. Internal seminal vesicle large, oval, curved. Pars prostatica oval vesicular, lined with anuclear cell-like bodies. Ejaculatory duct thick-walled, muscular, long, complexly folded. Genital atrium small. Genital pore closely sinistral to antero-sinistral to ventral sucker.

Ovary multilobate (about 14–20 lobes), immediately pre-testicular. Seminal receptacle saccular, dorsal to anterior testis. Laurer’s canal not detected. Mehlis’ gland dorsal to ovary. Uterus between ovary and ventral sucker, intracaecal. Eggs tanned, operculate. Vitellarium follicular, in extensive dorsal and ventral fields, from level of ventral sucker to posterior extremity; fields confluent at level of testes and in post-testicular region.

Excretory pore terminal; excretory vesicle narrow posteriorly, widens abruptly and reaches at least to posterior testes.

### Remarks

Several species of *Diploproctodaeum* are found in *Platax* spp., namely *D. plataxi* Mamaev, 1970, *D. rutellum* (Mamaev, 1970) and *D. tsubameuo* Bray & Cribb, 2003; all three species have caeca abutting the body-wall and are often described as having ani (Mamaev, 1970; Bray & Cribb, 2003a). Other lepocreadiid species from *Platax* spp., such as *Deraiotrema platacis* Machida, 1982, *Neomultitestis palauensis* Machida, 1982 and *N. aspidogastriformis* Bray & Cribb, 2003 are also described as having ani or the appearance of ani (Machida, 1982; Bray & Cribb, 2003a).

Phylogenetic analysis of the 28S dataset showed that this species does not form a strongly-supported clade with any particular clade of lepocreadiids. The new species was sister to a clade including *Neopreptetos arusettae* Machida, 1982, *Multitestis magnacetabulum* Mamaev, 1970 and *Neomultitestis aspidogastriformis*, the latter two of which are *Platax*-infecting species; however, nodal support for this relationship was poor. The new species was not closely related to species of other genera which have similar caecal terminations, namely *Diploproctodaeum*, *Bianium* and *Pelopscreadium*.

**Genus** ***Multitestis*** **Manter, 1931**

***Multitestis magnacetabulum*** **Mamaev, 1970**

*Type-host*: *Platax orbicularis* (Forsskål) (first host listed) (Perciformes: Ephippidae), orbicular batfish.

*Type-locality*: Gulf of Tonkin.

New records:

*Host*: *Platax teira* (Forsskål) (Perciformes: Ephippidae), longfin batfish.

*Locality*: Four Beacons, Moreton Bay (27°10′S, 153°21′E).

*Site in host*: Intestine.

*Voucher material*: Six voucher specimens QM G237262–7, three NHMUK 2018.3.26.9–11.

*Representative DNA sequences*: ITS2 rDNA, two replicates (one in GenBank MH157061); 28S rDNA, one sequence (GenBank MH157071).

*New measurements*: Supplementary Table S2.

### Remarks

This is the first record of this species from Moreton Bay. Bray & Cribb (2003a) reported it from *Platax teira* off Heron Island and Bray et al. (2009) used sequences from that collection in their molecular study of the superfamily Lepocreadioidea. 28S sequence data generated from new collections from Moreton Bay differed from the Heron Island specimens (GenBank FJ788485) by a single base. A single base difference is consistent with the minor geographical variation found between these locations for other trematodes (e.g. Cutmore et al., 2016; Brooks et al., 2017); however, given that this single base difference (an A to T transversion) is within the in the first 15 bases of the start of the sequence, and that this base position is an A in all other taxa included in the analysis, we predict that the difference in FJ788485 is a sequencing misread. This species has also been reported from the same host in the waters off New Caledonia by Bray & Justine (2012).

**Genus** ***Neomultitestis*** **Machida, 1982**

***Neomultitestis aspidogastriformis*** **Bray & Cribb, 2003**

*Type-host*: *Platax teira* (Forsskål) (Perciformes: Ephippidae), longfin batfish.

*Type-locality*: Off Heron Island, Queensland, Australia.

New records

*Host*: *Platax teira*.

*Locality*: Four Beacons, Moreton Bay (27°10′S, 153°21′E).

*Site in host*: Intestine.

*Voucher material*: One voucher specimen lodged in the QM G237268.

*Representative DNA sequences*: ITS2 rDNA, one sequence (GenBank MH157062); 28S rDNA, one sequence (GenBank MH157072).

*New measurements*: Supplementary Table S2.

### Remarks

Bray & Cribb (2003a) reported this species from *P*. *teira* off Heron Island, and Bray et al. (2009) used 28S rDNA sequences from that collection in their molecular study of the superfamily Lepocreadioidea. This is the first report of *N. aspidogastriformis* from Moreton Bay. New 28S data generated from Moreton Bay specimens were identical to those of this species off Heron Island (GenBank FJ788489).

**Genus** ***Opechona*** **Looss, 1907**

***Opechona austrobacillaris*** **Bray & Cribb, 1998**

*Type-host*: *Pomatomus saltatrix* (Linnaeus), tailor (Perciformes: Pomatomidae).

*Type-locality*: Off South Mole, Fremantle, Western Australia.

New material:

*Host*: *Pomatomus saltatrix*.

*Locality*: Off Garden Island, Moreton Bay (27°36′S, 153°20′E).

*Site in host*: Intestine.

*Voucher material*: Two specimens in the QM G237269–70, one in the NHMUK 2018.3.26.3.

*Representative DNA sequences*: ITS2 rDNA, two replicates (one in GenBank MH157063); 28S rDNA, one sequence (GenBank MH157073).

*New measurements*: Supplementary Table S2.

### Remarks

This is the first report of this species from Moreton Bay. Although the type-locality is off Western Australia, the original description also reported and described this species from the eastern coast of Australia, off Iluka in New South Wales (Bray & Cribb, 1998). Our specimens from Moreton Bay are indistinguishable from those described by Bray & Cribb (1998), and we are confident that the new specimens are conspecific with those from off Fremantle and Iluka.

New 28S sequence data generated for *O*. *austrobacillaris* differs from those of *O. kahawai*, from *Arripis* sp. off Tasmania, by just a single base. Unfortunately, no ITS2 rDNA sequence data (a superior marker for species delineation) are available for the Tasmanian species. Bray & Cribb (2003b) distinguished these two species by the sucker-ratio and the pseudoesophagus/oesophagus length ratio, and by the forebody being proportionally much longer in *O. kahawai* (40–44 *vs* 28–35% of body length) (Supplementary Table S2; Bray & Cribb, 1998). Given the minor genetic differences, the relationship between these two morphologically distinct forms warrants further study. Phylogenetic analysis of the 28S dataset showed these two species of *Opechona* to be most closely related to *Prodistomum keyam*; however, nodal support for this clade was poor.

**Genus** ***Prodistomum*** **Linton, 1910**

***Prodistomum keyam*** **Bray & Cribb, 1996**

*Type-host*: *Monodactylus argenteus* (Linnaeus) (Perciformes: Monodactylidae), silver moony.

*Type-locality*: Off Hope Island, Queensland, Australia.

New records:

*Host*: *Monodactylus argenteus*.

*Locality*: In Port of Brisbane Land Reclamation, Moreton Bay (27°21′S, 153°11′E); off Amity, Moreton Bay (27°24′S, 153°26′E).

*Site in host*: Intestine.

*Voucher material*: Four specimens in the QM G237271–4, one in the NHMUK 2018.3.26.3.

*Representative DNA sequences*: ITS2 rDNA, three identical replicates (one in GenBank MH157064); 28S rDNA, one sequence (GenBank MH157074).

*New measurements*: Supplementary Table S2.

### Remarks

Bray & Cribb (1996) and Bray et al. (2009) reported this host/species combination in Moreton Bay. Bray et al. (2009) used sequences of this species from this host in Moreton Bay in their molecular study of the superfamily Lepocreadioidea. Molecular data from new specimens collected in this study were identical to those (FJ788493) from Bray et al. (2009). Phylogenetic analysis of the 28S dataset showed *P. keyam* to be most closely related to *Opechona austrobacillaris* and *O*. *kahawai*, but with low support (Fig. 2). Bray & Justine (2012) reported this species from the same host from the waters around New Caledonia.

**Family Gibsonivermidae n. fam.**

*Diagnosis*

Body elongate-oval, flattened. Tegument armed with small spines. Oral sucker subglobular, subterminal. Ventral sucker rounded, pre-equatorial. Prepharynx distinct. Pharynx oval. Oesophagus distinct. Intestinal bifurcation in mid-forebody. Caeca form uroproct at posterior extremity. Testes two, lobed to almost entire, tandem, slightly separated, in mid-hindbody. External seminal vesicle very elongate, tubular, coiled, reaches well into hindbody. Cirrus-sac long, attenuated, coiled proximally. Internal seminal vesicle tubular, coiled. Pars prostatica long, narrow. Ejaculatory duct elongate, muscular, expands distally. Genital atrium small. Genital pore dextrally submedian, ventral to pharynx. Ovary with 4–6 lobes, pretesticular, slightly separated from anterior testis. Seminal vesicle between ovary and anterior testis. Uterus pre-ovarian, intercaecal; lateral slings extend into forebody. Metraterm narrow. Vitellarium follicular; fields reach from anterior region of hindbody or ventral sucker to posterior extremity. Excretory vesicle I-shaped, reaches anterior testis. In intestine of marine teleosts.

*Type-genus*: *Gibsonivermis* Bray, Cribb & Barker, 1997.

*ZooBank registration*: To comply with the regulations set out in article 8.5 of the amended 2012 version of the *International Code of Zoological Nomenclature* (ICZN, 2012), details of the new family have been submitted to ZooBank. The Life Science Identifier (LSID) for Gibsonivermidae n. fam. is urn:lsid:zoobank.org:act:F50B45FB-6B41-44B4-8FD2-5042D1AD938F.

### Remarks

Bray et al. (1997), in proposing *Gibsonivermis*, stated that it is ‘not immediately clear to which subfamily this genus belongs’, and Bray & Cribb (2012) considered *Gibsonivermis* a genus ‘*incertae sedis* within the superfamily’ Lepocreadioidea and ‘too enigmatic to allow confident placement’. Barker et al. (1993) sequenced the D1 domain of the 28S ribosomal RNA gene of the type-species of this new taxon under its old name *Intusatrium berryi* Gibson, 1987 but did not apparently submit the sequence to GenBank (it is itemised in the paper). In the early days of the development of molecular studies, few digenean sequences were available. The tree produced by Barker et al. (1993) included two other lepocreadioids, *Gyliauchen* sp. (Gyliauchenidae) and *Tetracerasta blepta* Watson, 1984 (Aephnidogenidae), which clustered with *Gibsonivermis*, but they stated that ‘evidence for the monophyly of the two lepocreadiids [*Tetracerasta* and *Gibsonivermis*] was weak’. The molecular phylogeny inferred from 28S data reported here confirms that *Gibsonivermis* does not belong to any of the six accepted lepocreadioid families (i.e. Lepocreadiidae, Aephnidiogenidae, Enenteridae, Gorgocephalidae, Gyliauchenidae, Lepidapedidae; see Bray & Cribb, 2012), constituents of which all form strongly supported clades. It is distinct enough, both morphologically and genetically, to warrant the proposal of a new family. In the current analyses, the Gibsonivermidae was sister to the Lepidapedidae, with branch lengths between the two families similar to those found between the Lepocreadiidae and Aephnidiogenidae, and the Enenteridae and Gyliauchenidae, indicative of a family level distinction.

**Fig. 3:**
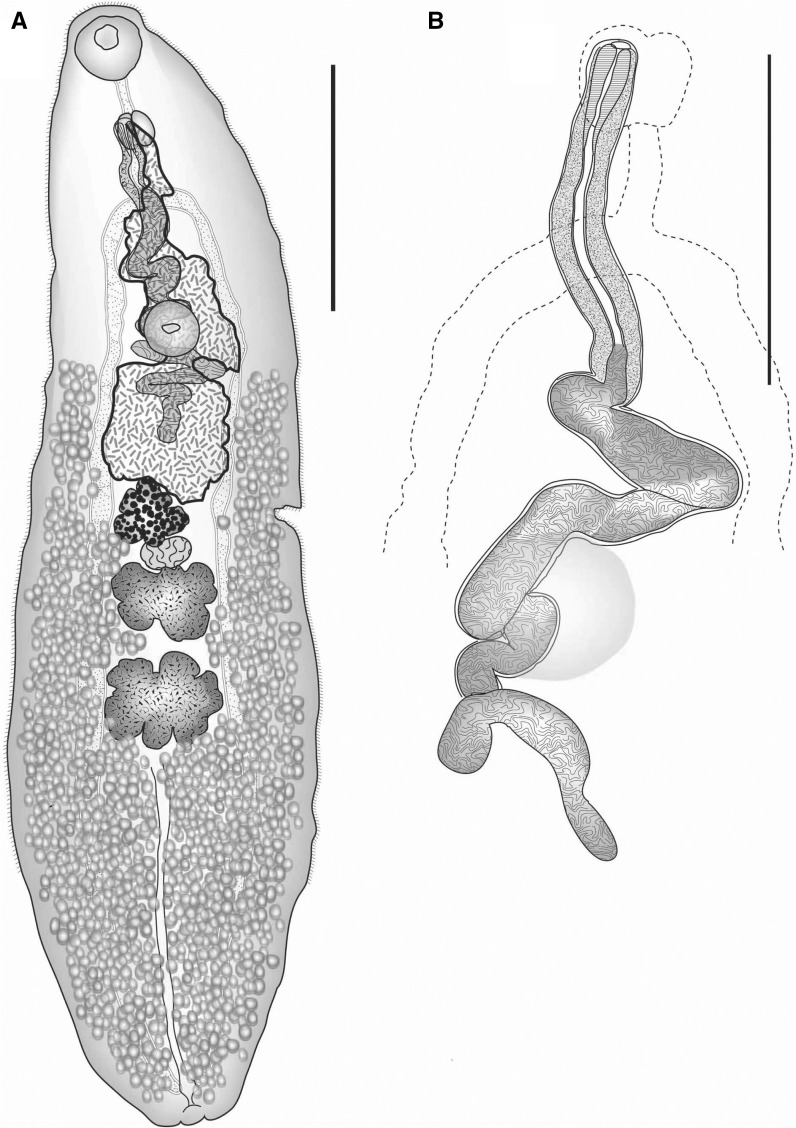
A, *Gibsonivermis berryi* (Gibson, 1987) ex *Sillago ciliata*. Holotype, ventral view, uterus in outline; B, *Gibsonivermis berryi* (Gibson, 1987) ex *Sillago analis*. Male terminal genitalia, with ventral sucker and gut in outline. *Scale-bars*: A, 1,000 μm; B, 500 μm

*Gibsonivermis berryi* (Gibson, 1987) Bray, Cribb & Barker, 1997 has several features very unusual for species within the superfamily, the most striking of which is the form of the male terminal genitalia (Fig. 3A, B). The cirrus-sac is elongate, narrow, coiled proximally and contains a long tubular coiled internal seminal vesicle, a long narrow pars prostatica and a muscular ejaculatory duct which widens distally (Gibson, 1987). The external seminal vesicle is long, tubular and coiled and merges into the internal seminal vesicle. Gibson (1987) described a constriction of the seminal vesicle as it enters the cirrus-sac but stated that it was only seen in sections. We have not been able to detect this constriction in whole-mounted worms. If it is always present, it is obscured by the folds of the seminal vesicle in the region dorsal to the ventral sucker in all the specimens we examined. This folding also usually obscures the precise posterior extent of the cirrus-sac wall. Other distinguishing features, which are rare or absent in other lepocreadioids, include a uroproct and a significant proportion of the uterus in the forebody. At present, no other lepocreadioids appear to have characters in any way resembling those of specimens of *Gibsonivermis*.

The single species of *Gibsonivermis* is so far known only from Moreton Bay and off Heron Island on the southern Great Barrier Reef. Bray et al. (1999) summarised the knowledge of the parasites of the Sillaginidae and found that no ‘lepocreadiids’ were reported outside Australian waters, but that in this region a few unusual, apparently endemic, forms occurred, namely species of *Gibsonivermis*, *Austroholorchis* Bray & Cribb, 1997 and *Lepidapedella* Bray, Cribb & Pichelin, 1999. *Austroholorchis* is now known to be an aephnidiogenid (see below). *Lepidapedella* is an unusual worm, which likewise does not agree well with any lepocreadioid family, but shows no morphological similarities to *G. berryi*, and was placed in the Lepidapedidae by Bray & Cribb (2012). The species of endemic Australian lepocreadioid genera which are not reported from sillaginids include the lepocreadiids *Amphicreadium* Bray & Cribb, 2001, *Cliveus* Bray & Cribb, 1997 and *Rugocavum* Bray & Cribb, 1997, the lepidapedids *Harveytrema* Kruse, 1979 and *Scaphatrema* Bray & Cribb, 1997, and the unassigned *Paraneocreadium* Kruse, 1978 and *Jericho* Bray & Cribb, 1997 (Kruse, 1978, 1979; Bray & Cribb, 1997a, 2001, 2012). None of members of these genera exhibit any great similarity to *G. berryi*, although the single species of *Paraneocreadium* has some extension of the uterus into the forebody and the testes are lobed (Kruse, 1978, 1979; Bray & Cribb, 1997a). Considering the recognition that *Gibsonivermis* warrants a separate family-level status within the Lepocreadioidea, the phylogenetic status of these other distinctive, apparent “southern endemics”, is of great interest. Since 1999, only records of opecoelids and transversotrematids have been added to the known sillaginid digenean fauna (Aken’Ova, 2003; Aken’Ova et al., 2008; Cutmore et al., 2016).

**Genus** ***Gibsonivermis*** **Bray, Cribb & Barker, 1997**

***Gibsonivermis berryi*** **(Gibson, 1987) Bray, Cribb & Barker, 1997**

Syn. *Intusatrium berryi* Gibson, 1987

*Type-host*: *Sillago ciliata* Cuvier (Perciformes: Sillaginidae), sand whiting.

*Type-locality*: Deception Bay, off Moreton Bay.

New record:

*Host*: *Sillago ciliata*.

*Locality*: Off Dunwich, Moreton Bay (27°29′S, 153°23′E).

*Voucher specimens*: Six specimens in the QM G237291–6.

*Representative DNA sequences*: ITS2 rDNA, two replicates (one in GenBank MH157060); 28S rDNA, one sequence (GenBank MH157070).

### Remark

Gibson (1987) and Bray et al. (1997) reported this species from the golden-line whiting *Sillago analis* Whitley, *S*. *ciliata* and the trumpeter whiting *S. maculata* Quoy & Gaimard, (Perciformes: Sillaginidae) from Moreton Bay. ITS2 rDNA data were found to be identical for specimens of this species infecting *S*. *ciliata* from Moreton Bay and off Heron Island.

**Family Aephnidiogenidae Yamaguti, 1934**

**Genus** ***Austroholorchis*** **Bray & Cribb, 1997**

***Austroholorchis sprenti*** **(Gibson, 1987) Bray & Cribb, 1997**

Syn. *Holorchis sprenti* Gibson, 1987

*Type-host*: *Sillago maculata* Quoy & Gaimard (Perciformes: Sillaginidae), trumpeter whiting.

*Type-locality*: Deception Bay, Moreton Bay.

New records:

*Host*: *Sillago ciliata* Cuvier.

*Locality*: Off Dunwich, Moreton Bay (27°29′S, 153°23′E).

*Voucher material*: Seven specimens in the QM G237284–90.

*Representative DNA sequences*: ITS2 rDNA, four replicates (one in GenBank MH157065); 28S rDNA, one sequence (GenBank MH157075).

### Remarks

Gibson (1987) and Bray & Cribb (1997b) reported this species from *Sillago analis*, *S. ciliata* and *S. maculata* from Moreton Bay. Analyses of the 28S data generated during this study indicate that this species forms a strongly-supported clade with all other included aephnidiogenids. Within the aephnidiogenid clade, *A*. *sprenti* formed a strongly-supported clade with species of *Aephnidiogenes* Nicoll, 1915, *Holorchis* Stossich, 1901 and *Neolepocreadium* Thomas, 1960, sister to the two freshwater anguilliform-infecting species *Stegodexamene anguillae* Watson, 1984 and *Tetracerasta blepta* Watson, 1984.

## Phylogenetic results

Alignment of the 28S rDNA dataset (Table 2) yielded 1,299 characters (including indels). Deleted ambiguously aligned regions amounted to 49 bases (less than 4% of the alignment), resulting in a final dataset of 1,250 characters for phylogenetic analysis. Bayesian inference and maximum likelihood analyses of the 28S rDNA dataset resulted in phylograms with almost identical topologies (Fig. 2). Only the relationship between the specimens of *Diploproctodaeum monstrosum* and *Diploproctodaeum* cf. *monstrosum* and that between *Lepidapedoides angustus* Bray, Cribb & Barker, 1996 and *Preptetos caballeroi* Pritchard 1960 were different. The topology was almost identical (but expanded relative) to that found by Bray et al. (2009), in which all lepocreadioid taxa formed a strongly supported clade to the exclusion of cryptogonimid and apocreadiid outgroup taxa. The now seven accepted families each formed monophyletic clades, all of which were strongly supported; nodal support for relationships within the familial clades was lower, especially for those in the lepocreadiid clade. The type- and only species of the Gibsonivermidae formed a well-supported clade with the lepidapedids. Most genera for which there were more than one sequenced species included formed monophyletic clades (*Gorgocephalus* Manter, 1966, *Holorchis*, *Hypocreadium* Ozaki, 1936, *Lepidapedon* Stafford, 1904, *Opechona*, *Paragyliauchen* Yamaguti, 1934 and *Proenenterum* Manter, 1954), but several formed notably polyphyletic assemblages (*Bianium*, *Diploproctodaeum* and *Preptetos*).

**Table 2:** Collection data and GenBank accession numbers for lepocreadioid species analysed in this study

Species	Host	GenBank ID	References
**Lepocreadioidea**			
**Aephnidiogenidae Yamaguti, 1934**			
*Aephnidiogenes major* Yamaguti, 1934	*Diagramma pictum labiosum* (Macleay)	FJ788468	Bray et al. (2009)
*Austroholorchis sprenti* (Gibson, 1987)	*Sillago ciliata* Cuvier	MH157075	Present study
*Holorchis castex* Bray & Justine, 2007	*Diagramma pictum pictum* (Thunberg)	FJ788476	Bray et al. (2009)
*Holorchis gigas* Bray & Cribb, 2007	*Plectorhinchus chrysotaenia* (Bleeker)	FJ788477	Bray et al. (2009)
*Neolepocreadium caballeroi* Thomas, 1960	*Trachinotus blochii* (Lacépède)	FJ788488	Bray et al. (2009)
*Stegodexamene anguillae* Macfarlane, 1951	*Gobiomorphus cotidianus* McDowall	KF484005	Herrmann et al. (2014)
*Tetracerasta blepta* Watson, 1984	*Posticobia brazieri* (Smith)	FJ788494	Bray et al. (2009)
**Enenteridae Yamaguti, 1958**			
*Enenterum aureum* Linton, 1910	*Kyphosus vaigiensis* (Quoy & Gaimard)	AY222232	Olson et al. (2003)
*Koseiria xishaensis* Gu & Shen, 1983	*Kyphosus vaigiensis*	AY222233	Olson et al. (2003)
*Proenenterum ericotylum* Manter, 1954	*Aplodactylus arctidens* Richardson	FJ788499	Bray et al. (2009)
*Proenenterum isocotylum* Manter, 1954	*Aplodactylus arctidens*	FJ788500	Bray et al. (2009)
**Gibsonivermidae n. fam.**			
*Gibsonivermis berryi* (Gibson, 1987)	*Sillago ciliata*	MH157070	Present study
**Gorgocephalidae Manter, 1966**			
*Gorgocephalus kyphosi* Manter, 1966	*Kyphosus vaigiensis*	AY222234	Olson et al. (2003)
*Gorgocephalus yaaji* Bray & Cribb, 2005	*Kyphosus cinerascens* (Forsskål)	KU951489	Huston et al. (2016)
*Gorgocephalus* sp.	*Austrolittorina unifasciata* (Gray)	KU951485	Huston et al. (2016)
**Gyliauchenidae Fukui, 1929**			
*Affecauda annulata* Hall & Chambers, 1999	*Naso tuberosus* Lacépède	FJ788501	Bray et al. (2009)
*Paragyliauchen arusettae* Machida, 1984	*Pomacanthus sexstriatus* (Cuvier)	FJ788503	Bray et al. (2009)
*Paragyliauchen* sp.	*Centropyge bicolor* (Bloch)	FJ788502	Bray et al. (2009)
*Petalocotyle adenometra* Hall & Cribb, 2000	*Prionurus microlepidotus* Lacépède	FJ788504	Bray et al. (2009)
*Robphildollfusium fractum* (Rudolphi, 1819)	*Sarpa salpa* (Linnaeus)	FJ788505	Bray et al. (2009)
**Lepocreadiidae Odhner, 1905**			
*Bianium arabicum* Sey, 1996	*Lagocephalus lunaris* (Bloch & Schneider)	MH157076	Present study
*Bianium plicitum* (Linton, 1928)	*Torquigener pleurogramma* (Regan)	MH157066	Present study
*Clavogalea trachinoti* (Fischthal & Thomas, 1968)	*Trachinotus coppingeri* Günther	MH157067	Present study
*Diplocreadium tsontso* Bray, Cribb & Barker, 1996	*Balistoides conspicillum* (Bloch & Schneider)	FJ788472	Bray et al. (2009)
*Diploproctodaeum momoaafata* Bray, Cribb & Barker, 1996	*Ostracion cubicus* Linnaeus	FJ788474	Bray et al. (2009)
*Diploproctodaeum monstrosum* Bray, Cribb & Justine, 2010	*Arothron stellatus* (Anonymous)	FJ788473	Bray et al. (2009)
*Diploproctodaeum* cf. *monstrosum*	*Arothron hispidus* (Linnaeus)	MH157069	Present study
*Echeneidocoelium indicum* Simha & Pershad, 1964	*Echeneis naucrates* Linnaeus	FJ788475	Bray et al. (2009)
*Hypocreadium patellare* Yamaguti, 1938	*Balistoides viridescens* (Bloch & Schneider)	FJ788478	Bray et al. (2009)
*Hypocreadium picasso* Bray, Cribb & Justine, 2009	*Rhinecanthus aculeatus* (Linnaeus)	FJ788479	Bray et al. (2009)
*Hypocreadium toombo* Bray & Justine, 2006	*Pseudobalistes fuscus* (Bloch & Schneider)	FJ788480	Bray et al. (2009)
*Lepidapedoides angustus* Bray, Cribb & Barker, 1996	*Epinephelus cyanopodus* (Richardson)	FJ788482	Bray et al. (2009)
*Lepotrema clavatum* Ozaki, 1932	*Acanthochromis polyacanthus* (Bleeker)	FJ788483	Bray et al. (2009)
*Lobatocreadium exiguum* (Manter, 1963)	*Pseudobalistes fuscus*	FJ788484	Bray et al. (2009)
*Mobahincia teirae* n. g., n. sp.	*Platax teira* (Forsskål)	MH157068	Present study
*Multitestis magnacetabulum* Mamaev, 1970	*Platax teira*	MH157071	Present study
*Neohypocreadium dorsoporum* Machida & Uchida, 1987	*Chaetodon flavirostris* Günther	FJ788487	Bray et al. (2009)
*Neomultitestis aspidogastriformis* Bray & Cribb, 2003	*Platax teira*	MH157072	Present study
*Neopreptetos arusettae* Machida, 1982	*Pomacanthus sexstriatus*	FJ788490	Bray et al. (2009)
*Opechona austrobacillaris* Bray & Cribb, 1998	*Pomatomus saltatrix* Linnaeus	MH157073	Present study
*Opechona kahawai* Bray & Cribb, 2003	*Arripis trutta* (Forster)	FJ788491	Bray et al. (2009)
*Pelopscreadium spongiosum* (Bray & Cribb, 1998)	*Ostracion cubicus*	FJ788469	Bray et al. (2009)
*Preptetos caballeroi* Pritchard, 1960	*Naso vlamingii* (Valenciennes)	AY222236	Olson et al. (2003)
*Preptetos trulla* (Linton, 1907)	*Ocyurus chrysurus* (Bloch)	AY222237	Olson et al. (2003)
*Prodistomum keyam* Bray & Cribb, 1996	*Monodactylus argenteus* (Linnaeus)	MH157074	Present study
**Lepidapedidae Yamaguti, 1958**			
*Bulbocirrus aulostomi* Yamaguti, 1965	*Aulostomus chinensis* (Linnaeus)	FJ788470	Bray et al. (2009)
*Intusatrium robustum* Durio & Manter, 1968	*Bodianus perditio* (Quoy & Gaimard)	FJ788481	Bray et al. (2009)
*Lepidapedon beveridgei* Campbell & Bray, 1993	*Coryphaenoides armatus* (Hector)	AJ405263	Bray et al. (2009)
*Lepidapedon desclersae* Bray & Gibson, 1995	*Mora moro* (Risso)	AJ405264	Bray et al. (1999)
*Lepidapedon discoveryi* Bray & Gibson, 1995	*Coryphaenoides armatus*	AJ405265	Bray et al. (1999)
*Lepidapedon elongatum* (Lebour, 1908)	*Gadus morhua* Linnaeus	AJ405266	Bray et al. (1999)
*Lepidapedon gaevskayae* Campbell & Bray, 1993	*Coryphaenoides armatus*	AJ405267	Bray et al. (1999)
*Lepidapedon rachion* (Cobbold, 1858)	*Gadus morhua*	AJ405260	Bray et al. (1999)
*Lepidapedon sommervillae* Bray & Gibson, 1995	*Coryphaenoides guentheri* (Vaillant)	AJ405268	Bray et al. (1999)
*Lepidapedon zubchenkoi* Campbell & Bray, 1993	*Coryphaenoides leptolepis* Günther	AJ405269	Bray et al. (1999)
*Myzoxenus insolens* (Crowcroft, 1945)	*Notolabrus tetricus* (Richardson)	FJ788486	Bray et al. (2009)
*Neolepidapedon smithi* Bray & Gibson, 1989	*Mora moro*	AJ405270	Bray et al. (1999)
*Postlepidapedon opisthobifurcatum* (Zdzitowiecki, 1990)	*Muraenolepis marmorata* Günther	KY497957	Sokolov et al. (2018)
*Postlepidapedon uberis* Bray, Cribb & Barker, 1997	*Choerodon venustus* (De Vis)	FJ788492	Bray et al. (2009)
*Profundivermis intercalarius* Bray & Gibson, 1991	*Coryphaenoides armatus*	AJ405271	Bray et al. (1999)
**Outgroup taxa**			
**Apocreadiidae Skrjabin, 1942**			
*Homalometron armatum* (MacCallum, 1895)	*Lepomis microlophus* (Günther)	AY222241	Olson et al. (2003)
*Neoapocreadium splendens* Cribb & Bray, 1999	*Scolopsis monogramma* (Cuvier)	AY222242	Olson et al. (2003)
*Paraschistorchis zancli* (Hanson, 1953)	*Zanclus cornutus* (Linnaeus)	AY222240	Olson et al. (2003)
**Cryptogonimidae Ward, 1917**			
*Adlardia novaecaledoniae* Miller, Bray, Goiran, Justine & Cribb, 2009	*Nemipterus furcosus* (Valenciennes)	FJ788496	Bray et al. (2009)
*Caecincola parvulus* Marshall & Gilbert, 1905	*Micropterus salmoides* (Lacépède)	AY222231	Olson et al. (2003)

## Discussion

Our phylogenetic hypotheses are inferred from the phylogram generated from the 28S rDNA dataset, but all are supported by morphology. This phylogram includes the sequences used by Bray et al. (2009) and Bray & Cribb (2012) in their reviews of the phylogeny and systematics of lepocreadioids and allows us to set the Moreton Bay worms in context. The uncontroversial results in the tree are the finding of identical sequences for *Neomultitestis aspidogastriformis* and *Clavogalea trachinoti* from off Heron Island and in Moreton Bay and the near identical sequences for *Multitestis magnacetabulum* from the same localities; species of several other trematode families have been shown to be genetically identical between the Great Barrier Reef and Moreton Bay (Brooks et al., 2017; Yong et al., 2018). More controversial is the close molecular similarity of *Opechona austrobacillaris* from Moreton Bay and *O. kahawai* from Tasmanian waters, which brings into question the status of these forms. Although morphologically similar, they do appear to be readily distinguishable. *Prodistomum keyam*, *O. austrobacillaris* and *O. kahawai* are included in a moderately-supported clade, which is poorly resolved internally. *Preptetos trulla* (Linton, 1907) is in this clade and is clearly not placed in the correct genus given its distance from the type-species, *P. caballeroi* Pritchard, 1960. *Preptetos trulla*, *Prodistomum keyam*, *O. austrobacillaris* and *O. kahawai* are also similar morphologically.

Moreton Bay members of the similar, and controversially separated, genera *Bianium* and *Diploproctodaeum* formed a well-supported clade, but were internally poorly resolved and clearly need greater sampling, both of species and genes, for a convincing arrangement to emerge. The morphological characteristics that are currently used to separate these genera are evidently unreliable. The five-base difference in sequences between *D. monstrosum* (‘*Diploproctodaeum* sp.’ in Bray et al., 2009) from off Lizard Island and *D.* cf. *monstrosum* from Moreton Bay indicates that there are potentially two closely related species in Queensland waters. However, few specimens have been collected from either location, and currently there are not enough morphological or molecular data to justify the proposal of a new species; this complex needs further study.

Members of the Lepocreadiidae *sensu stricto* in Moreton Bay were divided into two major clades reflecting the findings reported by Bray et al. (2009), who labelled the clades as VII and VIII. Clade VII includes what might be considered ‘typical’ lepocreadiids, mostly occurring in shallow water and, as far as is known, with a gastropod first intermediate host. Clade VIII includes many species from reef fishes, especially tetraodontiform fishes, with just one resolved life-cycle which utilises a bivalve first intermediate host (Hassanine, 2006). The distribution of these clades in terms of their assemblage and the nature of their hosts is worthy of further exploration, but a much wider understanding of the genetic structuring of this diverse family is needed before such an analysis can be completed.

The new status of *Gibsonivermis*, as the type-genus for a monotypic family, has been argued above. This new status is consistent with the observations of Cribb & Bray (2011) that new trematode families are now principally recognised from among known taxa rather than as a result of completely new discoveries. We suspect that further genetic exploration of unique trematode taxa will likely lead to more families being proposed within the Lepocreadioidea.

## Electronic supplementary material

Below is the link to the electronic supplementary material.

Supplementary material 1 (PDF 110 kb)

## References

[CR1] Aken’Ova TO (2003). A new species of *Podocotyloides* Yamaguti, 1934 (Digenea: Opecoelidae) from a Western Australian temperate marine fish. Systematic Parasitology.

[CR2] Aken’Ova TO, Cribb TH, Bray RA (2008). Eight new species of *Macvicaria* Gibson and Bray, 1982 (Digenea: Opecoelidae) mainly from endemic temperate marine fishes of Australia. ZooKeys.

[CR3] Ankenbrand MJ, Keller A, Wolf M, Schultz J, Förster F (2015). ITS2 Database V: Twice as much. Molecular Biology and Evolution.

[CR4] Barker SC, Blair D, Garrett AR, Cribb TH (1993). Utility of the D1 domain of nuclear 28S rRNA for phylogenetic inference in the Digenea. Systematic Parasitology.

[CR5] Blasco-Costa I, Cutmore SC, Miller TL, Nolan MJ (2016). Molecular approaches to trematode systematics: ‘best practice’ and implications for future study. Systematic Parasitology.

[CR6] Bray RA, Jones A, Bray RA, Gibson DI (2005). Family Lepocreadiidae Odhner, 1905. Keys to the Trematoda.

[CR7] Bray RA, Cribb TH (1996). Two species of *Prodistomum* Linton, 1910 (Digenea: Lepocreadiidae) from marine fishes of Australia. Systematic Parasitology.

[CR8] Bray RA, Cribb TH (1997). Lepocreadiid (Digenea) species from members of the marine teleost family Cheilodactylidae from south-western Australia, including four new genera and five new species. Systematic Parasitology.

[CR9] Bray RA, Cribb TH (1997). The subfamily Aephnidiogeninae Yamaguti, 1934 (Digenea: Lepocreadiidae), its status and that of the genera *Aephnidiogenes* Nicoll, 1915, *Holorchis* Stossich, 1901, *Austroholorchis* n. g., *Pseudaephnidiogenes* Yamaguti, 1971, *Pseudoholorchis* Yamaguti, 1958 and *Neolepocreadium* Thomas, 1960. Systematic Parasitology.

[CR10] Bray RA, Cribb TH (1998). Lepocreadiidae (Digenea) of Australian coastal fishes: new species of *Opechona* Looss, 1907, *Lepotrema* Ozaki, 1932 and *Bianium* Stunkard, 1930 and comments on other species reported for the first time or poorly known in Australian waters. Systematic Parasitology.

[CR11] Bray RA, Cribb TH (2001). *Amphicreadium* n. g. (Digenea: Lepocreadiidae) from monacanthid fishes (Tetraodontiformes) from the coast of northern Tasmania. Systematic Parasitology.

[CR12] Bray RA, Cribb TH (2003). Lepocreadiidae (Digenea) from the batfish of the genus *Platax* Cuvier (Teleostei: Ephippidae) from the southern Great Barrier Reef, Queensland, Australia. Systematic Parasitology.

[CR13] Bray RA, Cribb TH (2003). New species of *Opechona* Looss, 1907 and *Cephalolepidapedon* Yamaguti, 1970 (Digenea: Lepocreadiidae) from fishes off northern Tasmania. Papers and Proceedings of the Royal Society of Tasmania.

[CR14] Bray RA, Cribb TH (2012). Reorganisation of the superfamily Lepocreadioidea Odhner, 1905 based on an inferred molecular phylogeny. Systematic Parasitology.

[CR15] Bray RA, Cribb TH, Barker SC (1997). *Postlepidapedon* Zdzitowiecki, 1993 and *Gibsonivermis* n. g. (Digenea: Lepocreadiidae) from fishes of the southern Great Barrier Reef, Australia, and their relationship to *Intusatrium* Durio & Manter, 1968. Systematic Parasitology.

[CR16] Bray RA, Cribb TH, Justine J-L (2010). *Diploproctodaeum* spp. (Digenea, Lepocreadiidae) in Australian and New Caledonian waters including two new species from Tetraodontiformes and new records of related species. Acta Parasitologica.

[CR17] Bray RA, Cribb TH, Justine J-L (2010). *Multitestis* Manter 1931 (Digenea: Lepocreadiidae) in ephippid and chaetodontid fishes (Perciformes) in the south-western Pacific Ocean and the Indian Ocean off Western Australia. Zootaxa.

[CR18] Bray RA, Cribb TH, Pichelin SP (1999). Two new species of lepidapedines (Digenea, Lepocreadiidae) from the King George whiting *Sillaginodes punctata* (Perciformes, Sillaginidae) from off Kangaroo Island, South Australia. Acta Parasitologica.

[CR19] Bray RA, Gibson DI (1990). The Lepocreadiidae (Digenea) of fishes of the north-east Atlantic: review of the genera *Opechona* Looss, 1907 and *Prodistomum* Linton, 1910. Systematic Parasitology.

[CR20] Bray RA, Justine J-L (2012). A review of the Lepocreadiidae (Digenea, Lepocreadioidea) from fishes of the waters around New Caledonia. Acta Parasitologica.

[CR21] Bray RA, Waeschenbach A, Cribb TH, Weedall GD, Dyal P, Littlewood DTJ (2009). The phylogeny of the Lepocreadioidea (Platyhelminthes: Digenea) inferred from nuclear and mitochondrial genes: implications for their systematics and evolution. Acta Parasitologica.

[CR22] Brooks X, Cribb TH, Yong RQ-Y, Cutmore SC (2017). A re-evaluation of diversity of the Aporocotylidae Odhner, 1912 in *Siganus fuscescens* (Houttuyn) (Perciformes: Siganidae) and associated species. Systematic Parasitology.

[CR23] Cribb TH, Anderson GR, Adlard RD, Bray RA (1998). A DNA-based demonstration of a three-host life-cycle for the Bivesiculidae (Platyhelminthes: Digenea). International Journal for Parasitology.

[CR24] Cribb TH, Bray RA (2010). Gut wash, body soak, blender and heat-fixation: approaches to the effective collection, fixation and preservation of trematodes of fishes. Systematic Parasitology.

[CR25] Cribb TH, Bray RA (2011). Trematode families and genera: have we found them all?. Trends in Parasitology.

[CR27] Cutmore SC, Diggles BK, Cribb TH (2016). *Transversotrema* Witenberg, 1944 (Trematoda: Transversotrematidae) from inshore fishes of Australia: description of a new species and significant range extensions for three congeners. Systematic Parasitology.

[CR28] Darriba D, Taboada GL, Doallo R, Posada D (2012). jModelTest 2: more models, new heuristics and parallel computing. Nature Methods.

[CR29] Edgar RC (2004). MUSCLE: multiple sequence alignment with high accuracy and high throughput. Nucleic Acids Research.

[CR30] Gibson DI (1987). Two new lepocreadiids (Digenea) from *Sillago* spp. (Pisces: Sillaginidae) in Australian waters. Journal of Natural History.

[CR31] Hafeezullah M (1970). Lepocreadid trematodes of marine fishes of India. Parasitology.

[CR32] Hassanine RME-S (2006). The life cycle of *Diploproctodaeum arothroni* Bray and Nahhas, 1998 (Digenea: Lepocreadiidae), with a comment on the parasitic castration of its molluscan intermediate host. Journal of Natural History.

[CR33] Herrmann KK, Poulin R, Keeney DB, Blasco-Costa I (2014). Genetic structure in a progenetic trematode: signs of cryptic species with contrasting reproductive strategies. International Journal for Parasitology.

[CR34] Huston DC, Cutmore SC, Cribb TH (2016). The life-cycle of *Gorgocephalus yaaji* Bray & Cribb, 2005 (Digenea: Gorgocephalidae) with a review of the first intermediate hosts for the superfamily Lepocreadioidea Odhner, 1905. Systematic Parasitology.

[CR35] ICZN (2012). International Commission on Zoological Nomenclature: Amendment of articles 8, 9, 10, 21 and 78 of the International Code of Zoological Nomenclature to expand and refine methods of publication. Bulletin of Zoological Nomenclature.

[CR36] Kearse M, Moir R, Wilson A, Stones-Havas S, Cheung M, Sturrock S (2012). Geneious Basic: an integrated and extendable desktop software platform for the organization and analysis of sequence data. Bioinformatics.

[CR37] Keller A, Schleicher T, Schultz J, Müller T, Dandekar T, Wolf M (2009). 5.8S-28S rRNA interaction and HMM-based ITS2 annotation. Gene.

[CR38] Kruse GOW (1978). Trematodes of marine fishes from South Australia. 1. *Paraneocreadium australiense* gen. et sp. n. (Lepocreadiidae). Journal of Parasitology.

[CR39] Kruse GOW (1979). Trematodes of marine fishes from South Australia. 4. *Harveytrema bisulcatum* gen. et sp. n. (Lepocreadiidae). Journal of Parasitology.

[CR40] Linton E (1928). Notes on trematode parasites of birds. Proceedings of the United States National Museum.

[CR41] Littlewood DTJ (1994). Molecular phylogenetics of cupped oysters based on partial 28S rRNA gene sequences. Molecular Phylogenetics and Evolution.

[CR42] Littlewood DTJ, Curini-Galletti M, Herniou EA (2000). The interrelationships of Proseriata (Platyhelminthes: Seriata) tested with molecules and morphology. Molecular Phylogenetics and Evolution.

[CR43] Littlewood DTJ, Rohde K, Clough KA (1997). Parasite speciation within or between host species? Phylogenetic evidence from site-specific polystome monogeneans. International Journal for Parasitology.

[CR44] Machida M (1982). Lepocreadiid trematodes from marine fishes of Palau. Proceedings of the Japanese Society of Systematic Zoology.

[CR45] Maddison, W. P., & Maddison, D. R. (2017). Mesquite: a modular system for evolutionary analysis. Version 3.01 http://mesquiteproject.org.

[CR46] Mamaev, Y. L. (1970). [Helminths of some commercial fishes in the Gulf of Tong King.] In: Oshmarin, P. G., Mamaev, Y. L. & Lebedev, B. I. (Eds), *Helminths of Animals of South-East Asia*. Moscow: Izdatel’stvo Nauka, pp. 127–190 (In Russian).

[CR47] Miller, M. A., Pfeiler, E., & Schwartz, T. (2010). Creating the CIPRES Science Gateway for inference of large phylogenetic trees. In: Proceedings of the Gateway Computing Environments Workshop (GCE), 14 Nov. 2010, New Orleans, LA, pp. 1–8.

[CR48] Morgan JA, Blair D (1995). Nuclear rDNA ITS sequence variation in the trematode genus *Echinostoma*: An aid to establishing relationships within the 37-collar-spine group. Parasitology.

[CR49] Olson PD, Cribb TH, Tkach VV, Bray RA, Littlewood DTJ (2003). Phylogeny and classification of the Digenea (Platyhelminthes: Trematoda). International Journal for Parasitology.

[CR50] Ronquist F, Teslenko M, van der Mark P, Ayres DL, Darling A, Höhna S (2012). MrBayes 3.2: efficient Bayesian phylogenetic inference and model choice across a large model space. Systematic Biology.

[CR51] Sey O (1996). Description of *Bianium arabicum* sp. n. (Trematoda, Lepocreadiidae) from the pufferfish, *Lagocephalus lunaris* (Bloch et Schneider, 1801) in Kuwait and a review of the genus *Bianium* Stunkard, 1930. Parasitologia Hungarica.

[CR52] Shen J-W, Tong Y-Y (1990). Studies on the digenetic trematodes of fishes from the Daya Bay (Trematoda). Acta Zootaxonomica Sinica.

[CR53] Snyder SD, Tkach VV (2001). Phylogenetic and biogeographical relationships among some Holarctic frog lung flukes (Digenea: Haematoloechidae). Journal of Parasitology.

[CR54] Sokolov SG, Khasanov FK, Gordeev II (2018). New data on the morphology and phylogenetic connections of *Postlepidapedon opisthobifurcatum* (Trematoda, Lepocreadioidea: Lepidapedidae), a parasite of Antarctic and sub-Antarctic fishes. Helminthologia.

[CR55] Stamatakis A (2014). RAxML Version 8: A tool for phylogenetic analysis and post-analysis of large phylogenies. Bioinformatics.

[CR56] Sun D, Bray RA, Yong RQ, Cutmore SC, Cribb TH (2014). *Pseudobacciger cheneyae* n. sp. (Digenea: Gymnophalloidea) from Weber’s chromis (*Chromis weberi* Fowler & Bean) (Perciformes: Pomacentridae) at Lizard Island, Great Barrier Reef, Australia. Systematic Parasitology.

[CR57] Yong RQ-Y, Cutmore SC, Jones MK, Gauthier ARG, Cribb TH (2018). A complex of the blood fluke genus *Psettarium* (Digenea: Aporocotylidae) infecting tetraodontiform fishes of east Queensland waters. Parasitology International.

