# Supplementary material for: Lepocreadiidae Odhner, 1905 and Aephnidiogenidae Yamaguti, 1934 (Digenea: Lepocreadioidea) of fishes from Moreton Bay, Queensland, Australia, with the erection of a new family and genus
Source: Syst Parasitol. 2018 May 31;95(6):479–98. doi: 10.1007/s11230-018-9803-3 (PMC5993846; doi:10.1007/s11230-018-9803-3)
Supplement: Supplementary file 1 — Supplementary material 1 (PDF 110 kb) [file 11230_2018_9803_MOESM1_ESM.pdf]

**Electronic supplementary material for**

# Lepocreadiidae Odhner, 1905 and Aephnidiogenidae Yamaguti, 1934 (Digenea: Lepocreadioidea) of fishes from Moreton Bay, Queensland, Australia, with the erection of a new family and genus


**Rodney A. Bray • Thomas H. Cribb • Scott C. Cutmore**

Rodney A. Bray (✉)

Department of Life Sciences, Natural History Museum, Cromwell Road, London SW7 5BD, UK

e-mail: rab@nhm.ac.uk

Thomas H. Cribb • Scott C. Cutmore

The University of Queensland, School of Biological Sciences, St Lucia, Queensland 4072, Australia

**Supplementary Table 1** Measurements and ratios of *Bianium arabicum*, *B. plictum* and *B. dayawanense*, original and from the literature.

| Species | *Bianium arabicum* | | | *Bianium 'plicitum'* | *Bianium dayawanense* | *Bianium plicitum* | |
|---|---|---|---|---|---|---|---|
| Host | *Lagocephalus lunaris* | | | *Lagocephalus lunaris* | *Lagocephalus lunaris* | *Torquigener squamicauda* | *Torquigener pleurogramma* |
| Locality | Moreton Bay | Kuwait Bay | New Caledonia | Madras | Daya Bay, China | Moreton Bay | Moreton Bay |
| *n* | 3 | 36 | 15§ | | 4 | 3 | 1 [a] |
| Source | This study | Sey (1996) | Bray & Justine (2010) | Hafeezullah (1970)[c] | Shen & Tong (1990) | This study | This study |
| Body | 934–1,420 × 369–429 (1,251 × 403) | 650 [790 [b]]–1,025 × 300–380 | 852–1,225 × 263–416 (1,031 × 349) | 1,035 × 352 | 1,343 [1,586 [b]]–1,887 × 391–425 | 1,099–1,427 × 552–797 (1,281 × 688) | – |
| Forebody | 286–377 (345) | 249 [b] | 277–358 | 259 | 314 [b] | 356–480 | 505 |
| Pre-oral lobe | 40–71 (55) | 47 [b] | 42–74 (325 × 58) | 30 | 34–102 | 28–75 (429 × 54) | 52 |
| Oral sucker | 95–138 × 114–148 (123 × 134) | 70–104 × 65–113 | 105–131 × 107–133 (116 × 122) | 105 × 105 | 85–153 × 85–153 | 163–184 × 181–215 (176 × 193) | 200 × 230 |
| Prepharynx | 0 | 0+ | 0 | 0 | 25–34 | 0 | 0 |
| Pharynx | 74–126 × 79–111 (105 × 100) | 65–95 × 65–78 | 89–126 × 86–135 (105 × 109) | 82 × 82 | 51–102 × 68–102 | 99–116 × 116–132 108 × (123) | 157 × 181 |
| Oesophagus | 19–28 (25) | 0+ | 12–22 (17) | 9 | 34 [29 [b]]–68 | 4–59 (36) | 19 |
| Intestinal bifurcation to ventral sucker | 46–68 (55) | 53 [b] | 27–62 (49) | 37 | 74 [b] | 37–126 (67) | 99 |
| Vitellarium to ventral sucker | 0–44 (15) | 11 [b] | 0–36 (4) | 0 | 0 [b] | 0–120 (52) | 133 |
| Ventral sucker | 83–111 × 110–121 (98 × 114) | 67–90 × 65–90 | 82–108 × 82–105 (97 × 96) | 88 × 96 | 102–136 × 102–136 | 172–199 × 171–215 (186 × 195) | 181 × 209 |
| Cirrus-sac | 252–315 × 122–141 (293 × 128) | 120–128 × 40–50 | 195–294 × 46–121 (215 × 97) | 217 × 67 | 238–340 × 68–153 | 271–354 × 74–109 (311 × 92) | 455× 162 |
| External seminal vesicle to ventral sucker | 172–251 (224) | – | 63–188 (135) | 150 | 248 [b] | 47–120 (76) | 249 |
| Ventral sucker to ovary | 117–240 (199) | 120 [b] | 100–154 (124) | 165 | 277 [b] | 18–83 (43) | 104 |
| Ovary | 111–143 × 175–206 (128 × 187) | 80–100 × 45–60 | 50–158 × 94–182 (100 × 132) | 75 × 127 | 187–221 × 170–272 | 96–161 × 160–222 (135 × 181) | – |
| Ovary to anterior testis | 0 | 0 | 0–70 (5) | 0 | 0 | 0 | – |

| Species | *Bianium arabicum* | | | *Bianium 'plicitum'* | *Bianium dayawanense* | *Bianium plicitum* | |
|---|---|---|---|---|---|---|---|
| Host | *Lagocephalus lunaris* | | | *Lagocephalus lunaris* | *Lagocephalus lunaris* | *Torquigener squamicauda* | *Torquigener pleurogramma* |
| Locality | Moreton Bay | Kuwait Bay | New Caledonia | Madras | Daya Bay, China | Moreton Bay | Moreton Bay |
| *n* | 3 | 36 | 15§ | | 4 | 3 | 1 [a] |
| Source | This study | Sey (1996) | Bray & Justine (2010) | Hafeezullah (1970)[c] | Shen & Tong (1990) | This study | This study |
| Anterior testis | 111–150 × 104–158 (135 × 130) | 85–104 × 88–130 | 86–163 × 92–143 (121 × 111) | 180 × 143 | 187–238 × 119–204 | 182–244 × 146–174 (213 × 164) | – |
| Distance between testes | 0 | 0 | 0 | 0 | 0 | 0–16 (7) | – |
| Posterior testis | 147–235 × 130–167 (198 × 151) | 104–130 × 85–130 | 101–194 × 76–131 (145 × 109) | 232 × 142 | 204–396 × 119–221 | 194–255 × 164–195 (230 × 176) | – |
| Post-testicular distance | 138–250 (202) | 115* | 135–253 (186) | 172 | 301 [b] | 230–334 (266) | – |
| Post-caecal distance | 0 | 0 | 0 | 0 | 0 | 0 | – |
| Eggs | 57–64 × 37–51 (60 × 43) | 59–65 × 34–36 | 58–65 × 26–39 (62 × 32) | – | 48–54 × 30–39 | 65–71 × 28–39 (68 × 33) | 61 × 35 |
| Width % | 29.4–39.5 (33.0) | 37.1–37.1 | 29.1–37.6 (33.8) | 34.0 | 22.5–29.1 | 50.2–55.8 (53.4) | – |
| Forebody % | 26.6–30.6 (27.9) | 31.5 [b] | 29.1–34.7 (31.7) | 25.0 | 19.8 [b] | 31.6–36.5 (33.5) | – |
| Sucker length ratio 1: | 0.75–0.87 (0.81) | 0.87–0.96 | 0.74–0.94 (0.84) | 0.84 | 0.88–1.20 | 1.02–1.08 (1.05) | 0.91 |
| Sucker width ratio 1: | 0.79–0.97 (0.86) | 0.80–1.00 | 0.71–0.83 (0.79) | 0.91 | 0.88–1.20 | 0.95–1.09 (1.01) | 0.91 |
| Oral sucker: pharynx width 1: | 1.27–1.45 (1.36) | 0.69–1.00 | 0.96–1.24 (1.13) | 0.78 | 0.76–0.80 | 1.51–1.63 (1.56) | 1.27 |
| Ventral sucker to ovary % | 12.5–17.2 (15.5) | 15.2 [b] | 9.03–14.2 (12.1) | 15.9 | 17.5 [b] | 1.67–5.80 (3.20) | – |
| External seminal vesicle to ventral sucker % of VS-Ovary | 104–148 (119) | – | 46–172 (110) | 104 | 89.5 [b] | 145–337 (216) | 238 |
| Post-testicular distance% | 14.8–17.9 (16.0) | 14.6 [b] | 15.7–22.9 (18.0) | 16.6 | 19.0 [b] | 17.7–23.4 (20.7) | – |
| Prepharynx % | 0 | 0 | 0 | 0 | 1.86–1.80 [b] | 0 | – |
| Oesophagus% | 1.97–1.99 (1.98) | 0 | 1.09–2.17 (1.62) | 0.87 | 2.53–3.60 [b] | 0.29–4.14 (2.85) | – |
| IB-VS % | 3.24–5.59 (4.56) | 6.71 [b] | 3.09–6.12 (4.76) | 6.12 | 4.67 [b] | 2.62–9.57 (5.19) | – |

| Species | *Bianium arabicum* | | | *Bianium 'plicitum'* | *Bianium dayawanense* | *Bianium plicitum* | |
|---|---|---|---|---|---|---|---|
| Host | *Lagocephalus lunaris* | | | *Lagocephalus lunaris* | *Lagocephalus lunaris* | *Torquigener squamicauda* | *Torquigener pleurogramma* |
| Locality | Moreton Bay | Kuwait Bay | New Caledonia | Madras | Daya Bay, China | Moreton Bay | Moreton Bay |
| *n* | 3 | 36 | 15§ | | 4 | 3 | 1 [a] |
| Source | This study | Sey (1996) | Bray & Justine (2010) | Hafeezullah (1970)[c] | Shen & Tong (1990) | This study | This study |
| Vitellarium to ventral sucker distance % | 0–4.66 (1.55) | 1.39 [b] | 0–3.93 (0.42) | 0 | 0 | 0–8.38 (3.91) | – |
| Ovary to anterior testis % | 0 | 0 | 0–6 (0) | 0 | 0 | 0 | – |
| Distance between testes % | 0 | 0 | 0 | 0 | 0 | 0–1.16 (0.53) | – |
| Cirrus-sac length % | 22.2–27.0 (23.9) | 12.5–18.5 | 17.9–25.9 (21.0) | 21.0 | 17.7–18.0 | 23.4–24.8 (24.3) | – |
| Pre-vitelline distance | 242–377 (330) | 233 [b] | 277–357 (321) | 255 | 370 [b] | 319–480 (377) | 372 |
| Pre-vitelline distance % | 25.9–26.6 (26.3) | 29.5 [b] | 29.1–33.9 (31.2) | 24.6 | 23.3 [b] | 23.2–36.5 (29.6) | – |
| Oesophagus % of forebody | 6.50–7.46 (7.13) | 0 | 3.63–6.91 (5.13) | 3.47 | 9.24 [b] | 0.80–13.0 (8.86) | 3.67 |
| IB-VS % of forebody | 12.2–18.3 (16.2) | 21.3 [b] | 9.83–17.8 (15.0) | 14.3 | 23.6 [b] | 8.30–26.24 (14.99) | 19.5 |
| Vitellarium to ventral sucker distance % of forebody | 0–15.24 (5.08) | 4.42 [b] | 0–11.3 (1.21) | 0 | 0 [b] | 0–26.50 (12.28) | 26.3 |

[a] The single specimen of *Bianium plictum* from *Torquigener pleurogramma* was a hologenophore.

[b] Measurement taken from illustration.

[c] All measurements from Hafeezullah (1970) taken from illustration.

§ including measurements of 3 recently collected specimens.

% = % of body-length where not otherwise noted; IB-VS = Intestinal bifurcation to ventral sucker distance.

Where length is followed by width, the two are separated by an '×'.

**Supplementary Table 2** Measurements and ratios of *Multitestis magnacetabulum*, *Neomultitestis aspidogastriformis*, *Opechona austrobacillaris* and *Prodistomum keyam*.

| Species | *Multitestis magnacetabulum* | *Neomultitestis aspidogastriformis* | *Opechona austrobacillaris* | *Prodistomum keyam* |
|---|---|---|---|---|
| Host | *Platax teira* | *Platax teira* | *Pomatomus saltatrix* | *Monodactylus argenteus* |
| Locality | Moreton Bay | Moreton Bay | Moreton Bay | Moreton Bay |
| *n* | 9 | 1 | 2 | 4 |
| Source | This study | This study | This study | This study |
| Body | 392–1,029 × 469–772 (618 × 606) | 733 × 889 | 3,043–4,238 × 340–472 | 506–675 × 169–209 (578 × 193) |
| Forebody | 194–282 (224) | 288 | 1,039–1,328 | 247–325 (287) |
| Pre-oral lobe | 2–22 (6) | 9 | 0 | 0–3 (1) |
| Oral sucker | 91–147 × 104–166 (112 × 135) | 136 × 179 | 278–376 × 224–243 | 47–55 × 50–56 (51 × 53) |
| Prepharynx | 0 | 0 | 78–100 | 33–67 (53) |
| Pharynx | 63–126 × 72–106 (87 × 88) | 101 × 111 | 105–132 × 102–113 | 44–48 × 31–36 (46 × 34) |
| Oesophagus + pseudoesophagus | – | – | 549–660 | – |
| Oesophagus | 0 | 0 | 52–55 | 19–37 (25) |
| Pseudoesophagus | – | – | 487–605 | – |
| Intestinal bifurcation to ventral sucker | 0–110 (57) | 115 | 57–60 | 88–135 (110) |
| Vitellarium to ventral sucker | 115–200 (156) | 176 | 181–236 | 99–110 (104) |
| Ventral sucker | 114–195 × 199–310 (156 × 235) | 212 × 525 | 155–191 × 141–175 | 49–59 × 55–63 (54 × 58) |
| Cirrus-sac | 166–353 × 58–128 (217× 85) | 318 × 135 | 326–402 × 79–111 | 96–108 × 43–47 (102 × 45) |
| External seminal vesicle to ventral sucker | – | – | 270–420 | – |
| Ventral sucker to ovary | 0 | 0 | 577–754 | 16–102 (41) |
| Ovary | 65–112 × 73–167 (89 × 113) | 111 × 121 | 143–176 × 129–159 | 46–67 × 43–50 (52 × 45) |
| Ovary to anterior testis | 0–49 (14) | 0 | 14–84 | 0 |
| Anterior testis | 167–298 × 133–266 (218 × 193) | 230 × 620 | 215–265 × 214–197 | 59–72 × 67–81 (66 × 73) |
| Distance between testes | 89–193 (133) | 0 | 2–42 | 0 |

| Species | *Multitestis magnacetabulum* | *Neomultitestis aspidogastriformis* | *Opechona austrobacillaris* | *Prodistomum keyam* |
|---|---|---|---|---|
| Host | *Platax teira* | *Platax teira* | *Pomatomus saltatrix* | *Monodactylus argenteus* |
| Locality | Moreton Bay | Moreton Bay | Moreton Bay | Moreton Bay |
| *n* | 9 | 1 | 2 | 4 |
| Source | This study | This study | This study | This study |
| Posterior testis | 174–316 × 142–255 (231 × 200) | 37 × 42 | 243–280 × 195–215 | 73–89 × 79–100 (80 × 85) |
| Post-testicular distance | 141–442 (230) | 246 | 626–1,084 | 60–82 (69) |
| Post-caecal distance | – | – | 13–27 | 44–55 (50) |
| Eggs | 43–54 × 23–31 (50 × 28) | 52 × 31 | 82–90 × 41–48 | 54–59 × 26–38 (57 × 31) |
| Width % | 75–120 (102) | 121 | 11.1–11.2 | 30.9–37.2 (33.6) |
| Forebody % | 27.4–49.4 (37.6) | 39.4 | 31.3–34.1 | 48.1–52.7 (49.7) |
| Sucker length ratio 1: | 1.26–1.65 (1.40) | 1.56 | 0.51–0.56 | 1.02–1.11 (1.07) |
| Sucker width ratio 1: | 1.59–1.91 (1.75) | 2.93 | 0.63–0.72 | 1.01–1.14 (1.09) |
| Oral sucker: pharynx width 1: | 1.26–1.83 (1.53) | 1.61 | 2.14–2.20 | 1.49–1.75 (1.60) |
| Ventral sucker to ovary % | 0 | 0 | 17.8–18.9 | 3.13–15.1 (6.59) |
| External seminal vesicle to ventral sucker % of VS-Ovary | – | – | 46.8–55.7 | – |
| Post-testicular distance % | 29.8–43.0 (36.5) | 33.6 | 20.6–25.6 | 8.94–13.7 (12.0) |
| Prepharynx % | 0 | 0 | 7.52–7.53 | 3.11–5.50 (4.23) |
| Oesophagus % | 0 | 0 | 15.1–17.6 | 17.5–22.5 (19.0) |
| Oesophagus: pseudoesophagus ratio 1: | – | – | 10.6–11.0 | |
| IB-VS % | 0–14.3 (9.12) | 15.8 | 1.41–1.87 | 16.2–20.3 (18.2) |
| Vitellarium to ventral sucker distance % | 16.1–45.5 (27.0) | 24.0 | 5.56–5.94 | 0 |
| Ovary to anterior testis % | 0–8.01 (1.70) | 0 | 0–1.99 | 0 |
| Distance between testes % | 16.1–26.0 (21.9) | 0 | 0–1.00 | 18.0–18.9 (18.5) |
| Cirrus-sac length % | 24.3–48.4 (35.9) | 43.5 | 9.49–10.7 | 145–215 (183) |
| Pre-vitelline distance | 15–116 (67) | 112 | 858–1,093 | 28.6–35.3 (31.6) |
| Pre-vitelline distance % | 3.86–17.3 (10.6) | 15.3 | 25.8–28.2 | 13.3–20.5 (18.2) |
| Oesophagus % of forebody | 0 | 0 | – | 5.90–11.4 (8.56) |
| Oesophagus + pseudoesophagus % of forebody | – | – | 49.7–52.9 | – |

| Species | *Multitestis magnacetabulum* | *Neomultitestis aspidogastriformis* | *Opechona austrobacillaris* | *Prodistomum keyam* |
|---|---|---|---|---|
| Host | *Platax teira* | *Platax teira* | *Pomatomus saltatrix* | *Monodactylus argenteus* |
| Locality | Moreton Bay | Moreton Bay | Moreton Bay | Moreton Bay |
| *n* | 9 | 1 | 2 | 4 |
| Source | This study | This study | This study | This study |
| IB-VS % of forebody | 0–40.2 (24.9) | 40.0 | 4.51–5.48 | 35.7–42.6 (38.1) |
| Vitellarium to ventral sucker distance % of forebody | 58.4–92.2 (70.5) | 61.1 | 17.4–17.7 | 33.0–41.5 (36.6) |

% = % of body-length where not otherwise noted; IB-VS = Intestinal bifurcation to ventral sucker distance.

Where length is followed by width, the two are separated by an ‘×’.
